# Supplementary material for: Molecular Mimicry Analyses Unveiled the Human Herpes Simplex and Poxvirus Epitopes as Possible Candidates to Incite Autoimmunity
Source: Pathogens. 2022 Nov 16;11(11):1362. doi: 10.3390/pathogens11111362 (PMC9696880; doi:10.3390/pathogens11111362)
Supplement: Supplementary file 1 [file pathogens-11-01362-s001.zip › pathogens-1940082-supplementary.pdf]

**Table S1:** Viral-human homologs proteins metabolic pathways enrichment analysis.

| S.No | Human Uniprot IDs | Homologous Virus IDs | Number of Pathways | Pathway Source | Pathway name | p-value |
|---|---|---|---|---|---|---|
| **1** | sp\|P00374 \|DYR_HUMAN | gb:ABD28857 | 9 | REACTOME | Translesion synthesis by Y family DNA polymerases bypasses lesions on DNA template | 0.001016 |
| | | | | WikiPathways | ATM Signaling | 0.001069 |
| | | | | REACTOME | Signaling by ERBB4 | 0.001123 |
| | | | | SMPDB | Cetuximab Action Pathway | 0.001225 |
| | | | | SMPDB | Gefitinib Action Pathway | 0.001225 |
| | | | | SMPDB | Panitumumab Action Pathway | 0.001225 |
| | | | | SMPDB | Trastuzumab Action Pathway | 0.001225 |
| | | | | Spike | DNA damage induced G1-S checkpoint | 0.001474 |
| **2** | sp\|P04183 \|KITH_HUMAN | gb:AXN75107 | 9 | KEGG | p53 signaling | 0.001004 |
| | | | | WikiPathways | miRNA Regulation of DNA Damage Response | 0.001004 |
| | | | | REACTOME | RNA Polymerase II Transcription | 0.001055 |
| | | | | REACTOME | Post-translational protein phosphorylation | 0.00108 |
| | | | | REACTOME | G2/M Transition | 0.001198 |
| | | gb:AST09487 | | Panther_Pathway | p53 | 0.001222 |
| | | | | Spike | DAPk family | 0.001222 |
| | | | | REACTOME | Generic Transcription Pathway | 0.001227 |
| | | | | PID | Validated targets of C-MYC transcriptional activation | 0.001634 |
| **3** | sp\|P04818 \|TYSY_HUMAN | gb:QCA43223 | 4 | KEGG | Epithelial cell signaling in Helicobacter pylori infection | 0.001163 |
| | | | | REACTOME | Immune System | 0.001186 |
| | | gb:BBA90853 | | PID | Beta5 beta6 beta7 and beta8 integrin cell surface interactions | 0.001209 |
| | | | | WikiPathways | Cells and Molecules involved in local acute inflammatory response | 0.001209 |
| **4** | sp\|P09341 \|GROA_HUMAN | gb:AEV80662 | 5 | PID | Signaling events mediated by Stem cell factor receptor (c-Kit) | 0.001001 |
| | | | | KEGG | Endocytosis | 0.001179 |
| | | | | REACTOME | Signal Transduction | 0.001231 |
| | | | | REACTOME | Innate Immune System | 0.00134 |
| | | | | KEGG | Epithelial cell signaling in Helicobacter pylori infection | 0.001706 |
| **5** | sp\|P19875 \|CXCL2_HUMAN | gb:AEV80661 | 9 | Panther_Pathway | p53_pathway_feedback_loops_2 | 0.001033 |
| | | | | WikiPathways | Neural Crest Cell Migration in Cancer | 0.001033 |

| | | | | | | |
|---|---|---|---|---|---|---|
| | | | | WikiPathways | TNF related weak inducer of apoptosis (TWEAK) Signaling | 0.001033 |
| | | | | KEGG | Transcriptional misregulation in cancer | 0.001072 |
| | | | | Panther_Pathway | PI_3-Kinase | 0.001083 |
| | | | | PID | Syndecan-1-mediated signaling events | 0.001083 |
| | | | | WikiPathways | Interleukin-11 Signaling | 0.001083 |
| | | | | REACTOME | Signal Transduction | 0.001191 |
| | | | | KEGG | Legionellosis | 0.001766 |
| **6** | sp\|P23921\|RIR1_HUMAN | gb:AXN75085 | 14 | REACTOME | p53-Dependent G1 DNA Damage Response | 0.001002 |
| | | | | REACTOME | p53-Dependent G1/S DNA damage checkpoint | 0.001002 |
| | | | | REACTOME | Cell Cycle, Mitotic | 0.001022 |
| | | | | PID | mTOR signaling | 0.001049 |
| | | gb:AST09466 | | REACTOME | G1/S DNA Damage Checkpoints | 0.001097 |
| | | | | SMPDB | Morphine Metabolism Pathway | 0.001123 |
| | | | | Spike | Response to DSB | 0.001123 |
| | | | | KEGG | MicroRNAs in cancer | 0.001159 |
| | | | | KEGG | Hepatitis C | 0.001218 |
| | | gb:AUL80484 | | WikiPathways | Integrated Breast Cancer | 0.001218 |
| | | | | WikiPathways | Pyrimidine metabolism and related diseases | 0.00127 |
| | | | | ACSN2 | PENTOSE_PHOSPHATE | 0.001426 |
| | | | | WikiPathways | Nucleotide Metabolism | 0.001591 |
| | | | | HumanCyc | superpathway of pyrimidine deoxyribonucleotides <i>de novo</i> biosynthesis | 0.001764 |
| **7** | sp\|P31350\|RIR2_HUMAN | gb:AZY90656 | 12 | KEGG | p53 signaling | 0.001006 |
| | | | | WikiPathways | miRNA Regulation of DNA Damage Response | 0.001006 |
| | | | | WikiPathways | Pyrimidine metabolism and related diseases | 0.001035 |
| | | | | ACSN2 | DNA_DAMAGE_RESPONSE | 0.001049 |
| | | | | KEGG | Melanoma | 0.001049 |
| | | | | BioCarta | regulation of cell cycle progression by plk3 | 0.001162 |
| | | gb:AST09433 | | REACTOME | TP53 Regulates Transcription of Genes Involved in G2 Cell Cycle Arrest | 0.001162 |
| | | | | WikiPathways | TGF-B Signaling in Thyroid Cells for Epithelial-Mesenchymal Transition | 0.001162 |
| | | | | NetPath | Fibroblast growth factor-1 | 0.001183 |

| | | | | | | |
|---|---|---|---|---|---|---|
| | | | | REACTOME | The role of GTSE1 in G2/M progression after G2 checkpoint | 0.001183 |
| | | | | WikiPathways | Nucleotide Metabolism | 0.001296 |
| | | | | HumanCyc | superpathway of pyrimidine deoxyribonucleotides <i>de novo</i> biosynthesis | 0.001438 |
| **8** | sp\|P35354\|PGH2_HUMAN | gb:AEV80548 | 16 | KEGG | Bacterial invasion of epithelial cells | 0.001091 |
| | | | | KEGG | Pancreatic cancer | 0.001091 |
| | | | | NetPath | Fibroblast growth factor-1 | 0.001091 |
| | | | | NetPath | IL6 | 0.001091 |
| | | | | REACTOME | The role of GTSE1 in G2/M progression after G2 checkpoint | 0.001091 |
| | | | | BioCarta | er associated degradation (erad) | 0.0011 |
| | | | | PID | Degradation of beta catenin | 0.0011 |
| | | | | REACTOME | TP53 Regulates Transcription of Genes Involved in G2 Cell Cycle Arrest | 0.0011 |
| | | | | REACTOME | Downstream signaling events of B Cell Receptor (BCR) | 0.001179 |
| | | | | ACSN2 | PI3K_AKT_MTOR | 0.001208 |
| | | | | REACTOME | Protein ubiquitination | 0.001224 |
| | | | | REACTOME | Regulation of TP53 Activity through Methylation | 0.001227 |
| | | | | REACTOME | Immune System | 0.001266 |
| | | | | KEGG | Kaposi sarcoma-associated herpesvirus infection | 0.001488 |
| | | | | INOH | Prostaglandin Leukotriene metabolism | 0.001649 |
| | | | | Panther_Pathway | Inflammation_mediated_by_chemokine_and_cytokine_signaling | 0.001848 |
| **9** | sp\|P49916\|DNLI3_HUMAN | gb:AAY97564<br>gb:AST09563 | 8 | PID | Class I PI3K signaling events | 0.001034 |
| | | | | REACTOME | Signaling by EGFR | 0.001034 |
| | | | | BioCarta | pertussis toxin-insensitive ccr5 signaling in macrophage | 0.00104 |
| | | | | KEGG | Nucleotide excision repair | 0.001103 |
| | | | | ACSN2 | MITOCHONDRIAL_GENES | 0.001159 |
| | | | | REACTOME | Transcriptional regulation by small RNAs | 0.001159 |
| | | | | IPAVS | Gq_activated_pathways_in_maladaptive_hypertrophy | 0.001174 |
| | | | | KEGG | Th17 cell differentiation | 0.001201 |

**Table S2:** Viral proteins homologous with human proteins sharing homologous epitopes.

| S. No | Virus Homolog ID | Virus Protein name | Human Homologs IDs | Human Protein name |
|---|---|---|---|---|
| **1** | gb:AXN75085 | ribonucleotide reductase large subunit [Akhmeta virus] | sp\|P23921\|RIR1_HUMAN | Ribonucleoside-diphosphate reductase large subunit |
| **2** | gb:AST09466 | ribonucleotide reductase large subunit [NY_014 poxvirus] | sp\|P23921\|RIR1_HUMAN | Ribonucleoside-diphosphate reductase large subunit |
| **3** | gb:AEV80548 | prostaglandin G/H synthase 2 [Cercopithecine betaherpesvirus 5] | sp\|P35354\|PGH2_HUMAN | Prostaglandin-G/H synthase 2 |
| **4** | gb:AAY97564 | DNA ligase [Monkeypox virus] | sp\|P49916\|DNLI3_HUMAN | DNA ligase 3 |
| **5** | gb:AST09563 | ATP-dependent DNA ligase [NY_014 poxvirus] | sp\|P49916\|DNLI3_HUMAN | DNA ligase 3 |
| **6** | gb:AZY90656 | CPXV051 protein [Cowpox virus] | sp\|P31350\|RIR2_HUMAN | Ribonucleoside-diphosphate reductase subunit M2 |
| **7** | gb:AST09433 | ribonucleotide reductase small subunit [NY_014 poxvirus] | sp\|P31350\|RIR2_HUMAN | Ribonucleoside-diphosphate reductase subunit M2 |
| **8** | gb:QCA43223 | ORF13 [Human alphaherpesvirus 3] | sp\|P04818\|TYSY_HUMAN | Thymidylate synthase |
| **9** | gb:BBA90853 | ORF70 [Human gammaherpesvirus 8] | sp\|P04818\|TYSY_HUMAN | Thymidylate synthase |
| **10** | gb:AQY16903 | MC152 [Molluscum contagiosum virus subtype 1] | sp\|Q9H2F3\|3BHS7_HUMAN | 3 beta-hydroxysteroid dehydrogenase type 7 |
| **11** | gb:ADZ29327 | NMDA receptor-like protein [Cowpox virus] | sp\|Q9HC24\|LFG4_HUMAN | Protein lifeguard 4 |
| **12** | gb:AZT86284 | MC066L [Molluscum contagiosum virus] | sp\|P07203\|GPX1_HUMAN | Glutathione peroxidase 1 |
| **13** | gb:AXN75107 | thymidine kinase [Akhmeta virus] | sp\|P04183\|KITH_HUMAN | Thymidine kinase, cytosolic |
| **14** | gb:AST09487 | thymidine kinase [NY_014 poxvirus] | sp\|P04183\|KITH_HUMAN | Thymidine kinase, cytosolic |
| **15** | gb:QCF48225 | interleukin-10 [Human gammaherpesvirus 4] | sp\|P22301\|IL10_HUMAN | Interleukin-10 |
| **16** | gb:ABD28857 | ORF2 [Human gammaherpesvirus 8 | sp\|P00374\|DYR_HUMAN | Dihydrofolate reductase |
| **17** | gb:AAY97032 | dUTPase [Monkeypox virus] | tr\|H0YNW5\|H0YNW5_HUMAN | Deoxyuridine 5'-triphosphate nucleotidohydrolase |
| **18** | gb:AUL80434 | CPXV049 protein [Vaccinia virus] | tr\|A0A0C4DGL3\|A0A0C4DGL3_HUMAN | Deoxyuridine 5'-triphosphate nucleotidohydrolase |
| **19** | gb:AUL80132 | CPXV044 protein [Vaccinia virus] | sp\|Q8IV08\|PLD3_HUMAN | 5'-3' exonuclease PLD3 |
| **20** | gb:AAY97407 | unknown [Monkeypox virus] | sp\|Q9HC24\|LFG4_HUMAN | Protein lifeguard 4 |

| | | | | |
|---|---|---|---|---|
| **21** | gb:AUL80484 | hypothetical protein [Vaccinia virus] | sp\|P23921\|RIR1_HUMAN | Ribonucleoside-diphosphate reductase large subunit |
| **22** | gb:AUL80431 | CPXV045 protein [Vaccinia virus] | tr\|A0A3B3ITT3\|A0A3B3IT T3_HUMAN | Monoglyceride lipase |
| **23** | gb:AEV80662 | chemokine vCXCL7 [Cercopithecine betaherpesvirus 5] | sp\|P09341\|GROA_HUMA N | Growth-regulated alpha protein |
| **24** | gb:AEV80661 | chemokine vCXCL7 [Cercopithecine betaherpesvirus 5] | sp\|P19875\|CXCL2_HUMA N | C-X-C motif chemokine 2 |

**Table S3:** The hydrogen bonding interaction between the top ranked virus-human molecular mimic epitope and HLA and TLR receptors residues. The interactions was identified by molecular docking of epitope within HLA and TLR4 receptors.

| **HLA Receptor (Chain A) interactions with Human-virus identical epitope (Chain B)** |
|---|
| ASN(84):A<-->PHE(1):B |
| TYR(79):A<-->ASP(4):B |
| ASN(15):A<-->CYS(5):B |
| THR(83):A<-->LEU(13):B |
| THR(83):A<-->ASP(15):B |
| **TLR Receptor (Chain A) interaction with Human-virus identical epitope (Chain B)** |
| CYS(585):A<-->ASP(6):B |
| GLU(586):A<-->CYS(7):B |
| GLU(586):A<-->LEU(8):B |
| LYS(595):A<-->CYS(1):B |
| VAL(620):A<-->CYS(1):B |
| ASN(624):A<-->LEU(2):B |

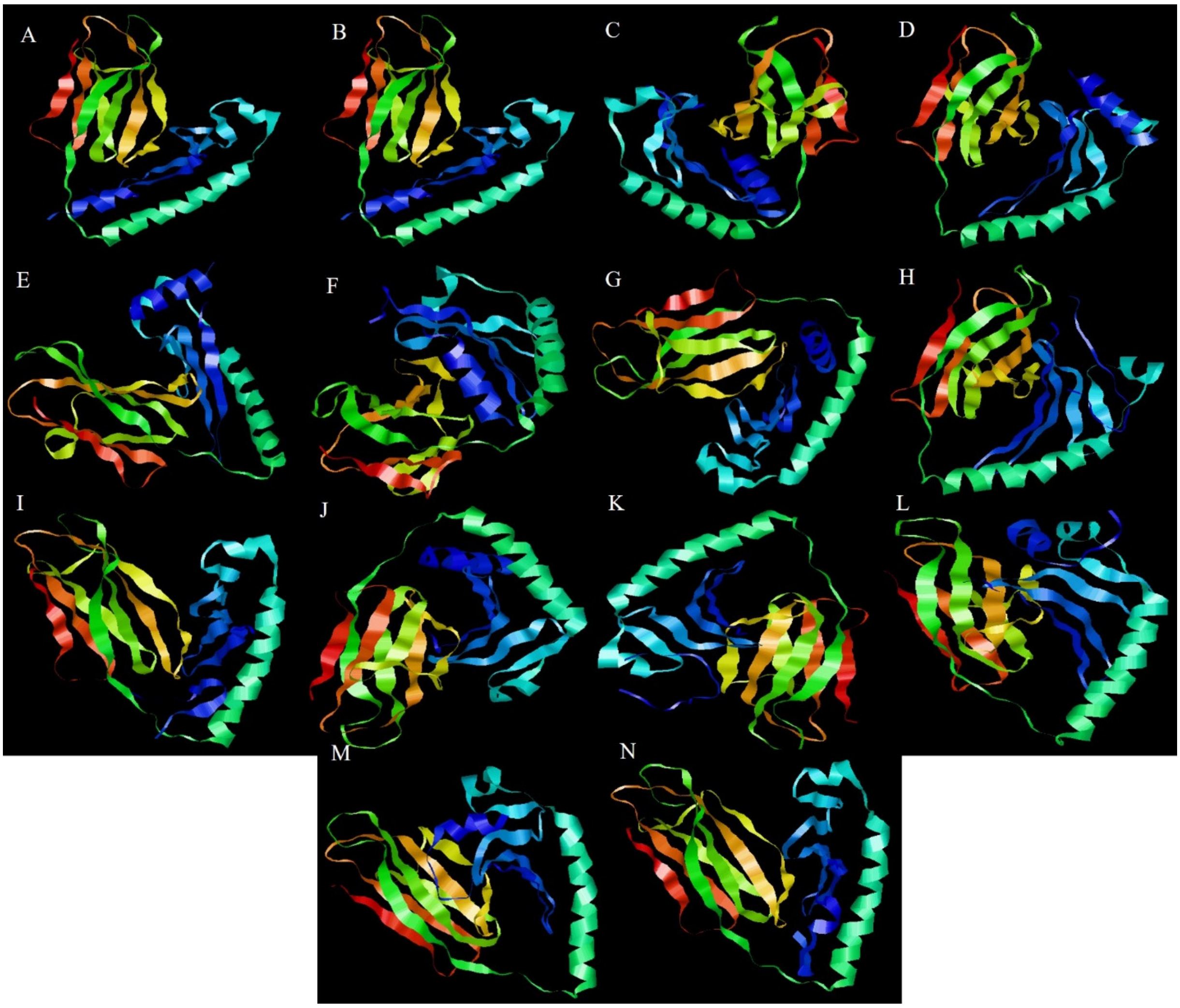


**Figure S1:** Molecular docking results of mimicking human peptides docked with HLA receptor.

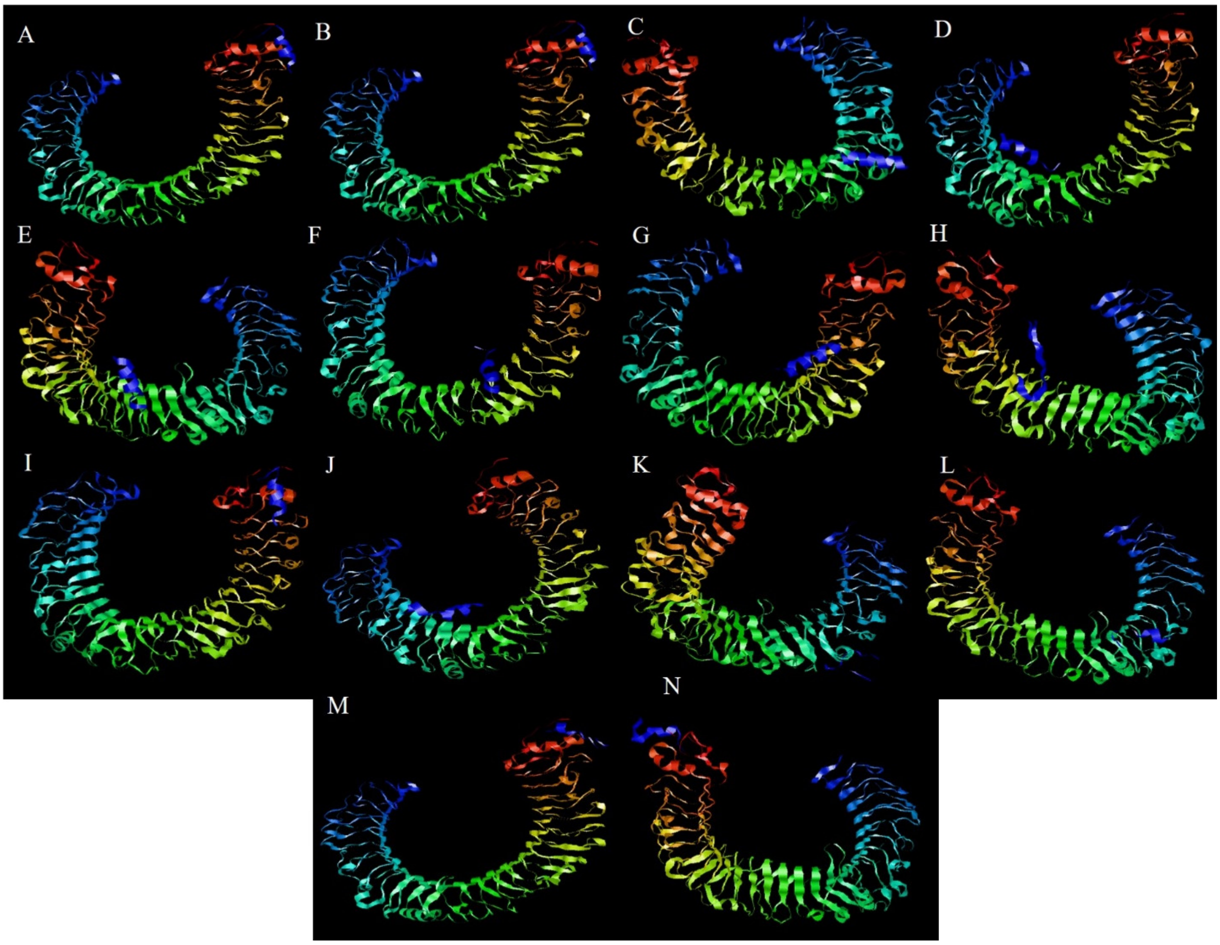


**Figure S2:** Molecular docking results of mimicking human peptides docked with TLR4 receptor.

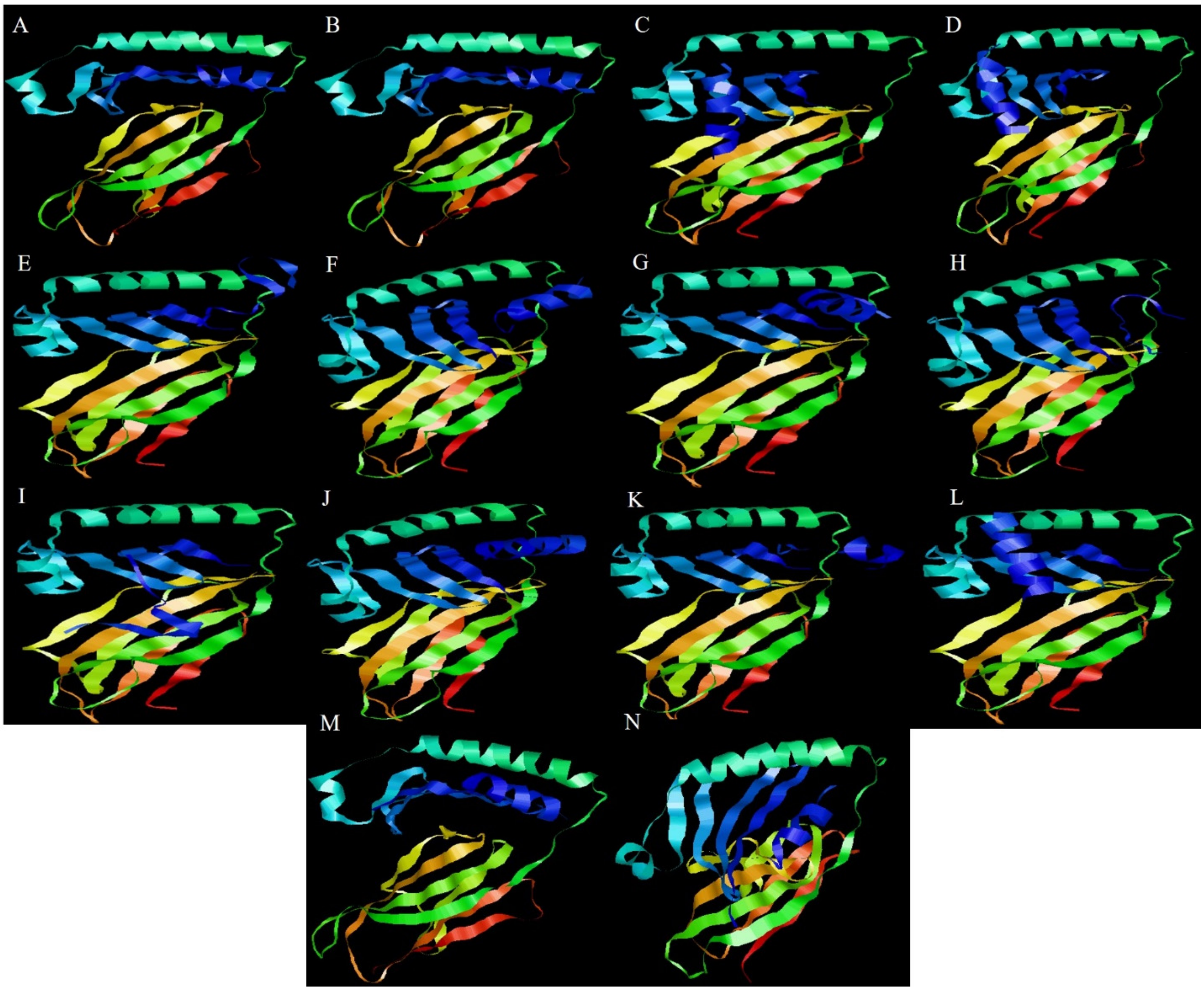


**Figure S3:** Molecular docking results of mimicking viral peptides docked with HLA receptor.

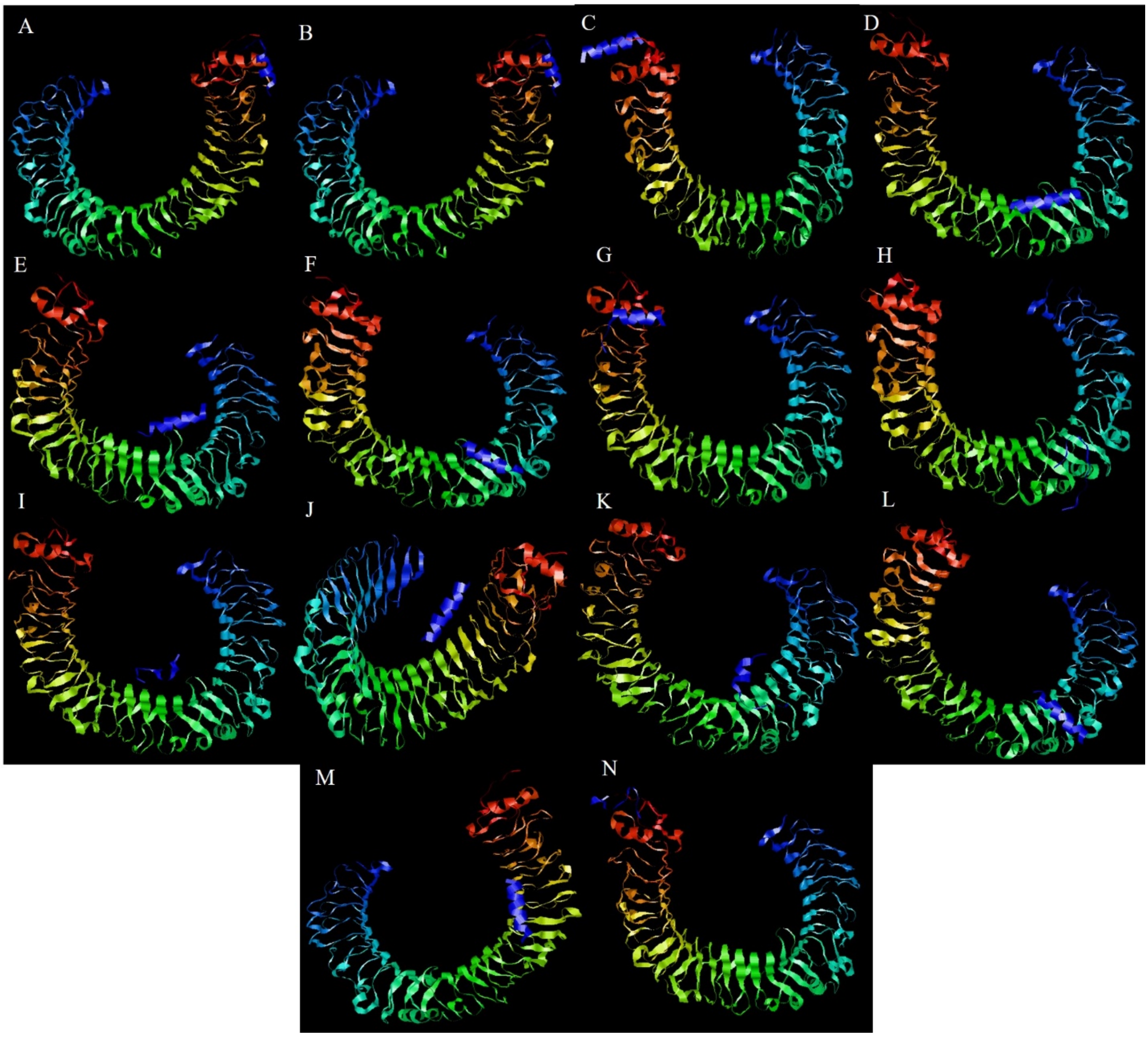


**Figure S4:** Molecular docking results of mimicking viral peptides docked with TLR4 receptor.
